# Assessing the work-related health and well-being of yoga and naturopathy professionals: A cross-sectional study from India

**DOI:** 10.1016/j.jaim.2024.101089

**Published:** 2025-05-09

**Authors:** Pradeep M.K. Nair, Karishma Silwal, Prakash Babu Kodali, Gulab Tewani

**Affiliations:** a Mirakle Integrated Health Centre, Pollachi, Tamilnadu, India; b Department of Naturopathy, Sant Hirdaram Medical College of Naturopathy & Yogic Sciences for Women, Bhopal, India; c Department of Public Health and Community Medicine, Central University of Kerala, Kasaragod, Kerala, India; d Sant Hirdaram Yoga and Nature Cure Hospital, Bhopal, India

**Keywords:** Occupational health, Quality of life, Health Personnel, Burnout, Workplace safety

## Abstract

**Background:** Health workers face significant hazards in their occupational settings. While many studies document the health risks of conventional healthcare workers, CAM health workers encounter unique challenges, including physical strain from repetitive tasks and psychosocial stressors from intensive patient interactions. Despite the widespread practice of yoga and naturopathy in India and globally, there is a lack of reports on the occupational health and well-being of yoga and naturopathy health workers. **Objectives:** This study aims to fill this gap by exploring the occupational health issues and well-being of yoga and naturopathy professionals, providing valuable insights to improve their work environments and support systems. **Methods:** A cross-sectional study was conducted in a yoga and naturopathy-based lifestyle medicine hospital in India. Participants included doctors, interns, therapists, drivers, and administrative, kitchen, and housekeeping staff with at least one year of work experience. Data on general well-being, quality of life, fatigue, and work-related burnout were collected using validated questionnaires. Data analysis involved univariate and multivariate approaches to compute prevalence and identify factors associated with burnout, pain, and occupational symptoms. **Results:** Among 138 participants, 68.1% were female, and 34.1% were therapists. Most participants were overweight or obese. High burnout levels were reported by 29%, with eye strain (41.3%) and sleep disturbances (26.8%) being prevalent occupational health issues. Females and those with up to six years of experience had higher odds of occupational symptoms and burnout. **Conclusions:** The majority perceived the workplace as safe, with no reports of sexual harassment. This study highlights the need for targeted interventions to improve the occupational health and well-being of yoga and naturopathy health workers. Future research should include larger, more diverse samples across multiple settings to validate and expand upon these findings.

## 1 Introduction

Occupational health is a critical area of public health that focuses on the prevention and management of work-related injuries, illnesses, and conditions [1]. In various work environments, employees encounter numerous physical, chemical, biological, and psychosocial hazards that can significantly affect their well-being. Health workers, in particular, are regarded as working in one of the most hazardous occupational settings. Many studies have documented the occupational health risks faced by healthcare workers [2,3]. However, the majority of studies reporting occupational health risks among healthcare workers focus on those working in conventional medical settings.

Health workers in complementary and alternative medicine (CAM) settings face unique challenges that differ significantly from those encountered by conventional health workers. One of the primary challenges is the physical strain associated with specific CAM practices. For instance, masseurs and physical therapists frequently perform repetitive tasks and maintain awkward postures for extended periods, leading to a higher risk of musculoskeletal disorders compared to conventional medical practitioners [4,5].

Additionally, CAM practitioners often have varied and sometimes more intensive patient interactions, as their therapeutic approaches are typically holistic and patient-centered, requiring more time and emotional investment in each session [6]. This increased patient interaction can lead to distinct psychosocial stressors, such as emotional exhaustion and burnout, which are less prevalent in conventional healthcare settings where patient interactions are often more standardized and time-limited.

India is one of the many countries that have regulated CAM practices and offer CAM services through registered medical practitioners. Yoga and naturopathy are among the largest CAM systems of medicine practiced worldwide, including in India [7]. Despite the widespread practice and regulation of yoga and naturopathy as major CAM systems in India and globally, there is a notable lack of reports on the occupational health, general well-being, and quality of life among yoga and naturopathy health workers.

Understanding the unique challenges faced by these practitioners is crucial for developing targeted interventions and policies that ensure their safety and enhance their overall quality of life. Furthermore, considering the role of yoga and naturopathy professionals as lifestyle medicine experts, understanding how well they maintain their own lifestyles is essential for promoting wellness as a priority among wellness providers. By examining their health practices and well-being, we can identify areas for improvement and support, ultimately fostering a culture of health and wellness that not only benefits practitioners but also enhances the quality of care they provide to their clients.

Therefore, this study aims to fill this gap by exploring the occupational health issues and well-being of yoga and naturopathy professionals, providing valuable insights to improve their work environments and support systems.

## 2 Methodology

### 2.1 Study design and settings

This study used a cross-sectional study design to evaluate the prevalence of occupational risk and hazards among the health workers from a private yoga and naturopathy-based lifestyle medicine hospital from India. The study was approved by the institutional ethics committee of the hospital (Approval no 1–2/SHMCNYS/P34). All the participants signed an informed consent before sharing their data.

### 2.2 Study participants

The study included both males and females, including doctors, interns, therapists, drivers, and administrative, kitchen, and housekeeping staff who were working in the study setting from September 2022 to December 2022. Participants with a minimum of one year of work experience in the study setting were considered eligible.

### 2.3 Outcome measures

#### 2.3.1 Work related health and well being

A face-to-face interview was conducted to assess the general well-being using a structured questionnaire among the study participants. The questions were first piloted among 15 participants and validated before the survey. The questionnaire consisted of variables under three domains: i) variables about the participants' demographic and occupational characteristics, ii) variables about the presence of occupational-related health ailments such as pain, sprain, strain, varicose veins, stiffness, any allergic and cold symptoms, sleep disturbances, digestive issues, and eyestrain, and iii) variables to investigate perceptions about the workplace, workplace safety, and the presence of any type of sexual harassment.

#### 2.3.2 Quality of life

Quality of life was measured using SF 12 health questionnaire [8] which assessed the physical and mental QoL using eight health domains such as general health, physical functioning, role physical, body pain, vitality, social functioning, role emotional, and mental health. The final score was summarized as physical component summary scores (PCS) and mental component summary scores (MCS).

#### 2.3.3 Fatigue

Chalder fatigue scale [9] was used to measure the severity of tiredness. It consists of 11 questions which are answered on a 4-point Likert scale. The global score can range from 1 to 33 with a higher score indicating more tiredness.

#### 2.3.4 Work-related burnout

Burnout is a term for an exhausted and pessimistic attitude toward work. Oldenburg burnout inventory (OLBI) [10], a 16-item self-report questionnaire was used to measure burnout that uses a five-point rating system, with one being “Strongly agree” and 4 being “Strongly disagree.” The total score can range from 16 to 64 with higher score suggesting more level of burnout.

#### 2.3.5 Data cleaning and analysis

The data from cross-sectional survey of 138 individuals working in naturopathy was obtained. Prior to the analysis the data was cleaned to identify and address any missing data, inconsistencies in data entry, and to transform variables to facilitate analysis. We did not find any specific missing data; the identified data entry errors were addressed by referring to the original survey questionnaires. The responses to Oldenburg Burnout Inventory, and Chadler Fatigue Scale were combined to develop a single composite score. Further, these composite scores were categorized into two categories i) low and ii) high, based on the class intervals. The PCS and MCS scores were computed as per the scoring indications of SF-12 scale. The composite scores of PCS and MCS were further categorized as low and high employing a cutoff score of 50.0.

Additionally, body pains including back pain, general body ache, knee pain, shoulder pain, were combined into a single variable “pain” coded as i) absent and ii) present. Similarly, all the occupational symptoms such as i) pain, ii) sprain/strain, iii) varicose veins, iv) general stiffness, v) allergic/contact dermatitis, vi) sleep disturbances, vii) digestive symptoms, viii) frequent cough and cold, and ix) eye strain were combined into a single variable “experienced any occupational symptoms”. Furthermore, blood pressure was categorized as normal/high based on the cutoff value of 140/90 mm Hg of mercury. Based on body mass index (BMI) an individual was categorized as underweight and normal (≤22.90), overweight (22.91–24.90), and obese (24.91 and above).

The data analysis was conducted employing univariate, and multivariate approaches. We computed the percentage prevalence and 95% confidence intervals (CI) for burnout, occupational symptoms, and pain. Binary logistic regression was employed to develop multi-variate models to identify the factors associated with burnout, pain, occupational symptoms, PCS and MCS among the study participants. The multi-variate models were adjusted for demographic and occupational characteristics. The model fit of the binary logistic regression was decided based on the p > 0.05 for the Hosmer-Lemeshow test. All the binary logistic regression models were found to have an acceptable model fit. The adjusted odds ratios (AOR) and 95% CI of AOR were computed.

## 3 Results

We surveyed a total of 138 individuals working in naturopathy settings in a naturopathy hospital in Central India. All the employees who qualified as per the inclusion criteria were surveyed. Among the participants 68.1% (n = 94) were females, and 34.1% (n = 48) were therapists. A majority (68.8%) were aged up to 45 years, and 52.2% (n = 72) had a work experience of up to seven years. Table 1 outlines the demographic and occupational characteristics of study sample.

**Table 1:** Demographic and occupational characteristics of study sample (n = 138).

Variables	Frequency (n)	Percentage (%)
**Age group**		
Up to 45 years	95	68.8
45 years and above	43	31.2
**Sex of the participant**		
Female	94	68.1
Male	44	31.9
**Designation**		
Therapists	48	34.8
Doctors and interns	36	26.1
Support Staff	54	39.1
**Daily working hours**		
Up to 8 h	105	76.1
Greater than 8 h	33	23.9
**Standing hours during work**		
Less than 4 h	59	42.8
Greater than 4 h	79	57.2
**Sitting hours**		
Less than 4 h	59	42.8
Greater than 4 h	79	57.2
**Break time at work**		
Up to 30 min per day	85	61.6
Greater than 30 min per day	53	38.4
**Years of work experience**		
Up to 6 years	72	52.2
7 years and above	66	47.8

We analysed the prevalent of health aliments and burnout among the study participants. It was found that close to 60% of the study participants were close to overweight or having obese BMI. It was found that 29% (95% CI = 21.8–36.9) had a high level of burnout. Among occupational health effects, it was found that 41.3% (95% CI = 33.3–49.6) experienced eye strain, and 26.8% (95% CI = 19.9–34.6) experienced sleep disturbances (see Table 2).

**Table 2:** Health ailments and occupational burnout among the study participants (n = 138).

Variables	Percentage (%)	95% CI
**Blood Pressure**		
Normal	87.7	81.5–92.5
High	12.3	7.5–18.5
**BMI**		
Underweight and normal	41.3	33.3–49.6
Over Weight	23.9	17.3–31.5
Obese	34.8	27.2–43.0
**Pain**		
Absent	29.0	21.8–36.9
Present	71.0	63.1–78.2
**Sprain/Strain**		
Absent	83.3	76.5–88.9
Present	16.7	11.1–23.5
**Varicose Veins**		
Absent	90.6	85.0–94.7
Present	9.4	5.3–15.0
**General Stiffness**		
Absent	73.9	66.2–80.8
Present	26.1	19.2–33.8
**Allergic/Contact dermatitis**		
Absent	83.3	76.5–88.9
Present	16.7	11.1–23.5
**Sleep disturbances**		
Absent	73.2	65.4–80.1
Present	26.8	19.9–34.6
**Digestive Symptoms**		
Absent	79.7	72.5–85.8
Present	20.3	14.2–27.5
**Frequent Cough and Cold**		
Absent	80.4	73.3–86.5
Present	19.6	13.5–26.7
**Eye Strain**		
Absent	58.7	50.4–66.7
Present	41.3	33.3–49.6
**CFS Score**		
Low	92.0	86.7–95.8
High	8.0	4.2–13.3
**Burn Out**		
Low	71.0	63.1–78.2
High	29.0	21.8–36.9

**PCS (Mean ± SD)**	47.3 (8.0)	
MCS **(Mean ± SD)**	51.6 (9.8)	

The data of PCS and MCS are mean (standard deviation); The data on health ailments and occupational burnout are expressed as percentage (95% CI).

The multivariate models revealed that females (AOR = 5.11, 95% CI = 1.33–19.72), Doctors and interns (AOR-15.88, 95% CI = 1.15–218.45), those with experience of working in naturopathy settings for up to six years (AOR = 5.67; 95% CI = 1.38–23.18) and obese (AOR = 0.21, 95% CI = 0.05–0.91) had significantly higher odds of having occupational symptoms. Similarly, females (2.91, 95% CI = 1.08–2.84) and therapists (AOR = 3.34, 95% CI = 1.12–10.09) have a greater odd of high levels of burnout. Additionally, females(4.52, 95% CI = 1.73–11.77), therapists(2.71, 95% CI = 1.01–7.30), doctors and interns(7.27, 95% CI = 1.45–36.36) exhibited higher odds of experiencing pain. Table 3 outlines the results of multivariate models. Majority of the participant's perception of the workplace environment is safe i.e. (95.65%). None of the participants experienced sexual harassment.

**Table 3:** Factors influencing occupational health of staff in naturopathy settings (n = 138).

Independent Variables	Burnout	Pain	Any occupational symptoms	PCS	MCS
**Age group**					
Up to 45 years	3.46 (0.94–12.81)	0.56 (0.18–1.80)	0.17 (0.04–0.77) ∗	0.87 (0.29–2.62)	1.90 (0.59–6.14)
46 years and above (ref)					
**Sex of the participant**					
Female	2.91 (1.08–7.84) ∗	4.52 (1.73–11.77) ∗∗	5.11 (1.33–19.72) ∗	0.55 (0.23–1.25)	0.37 (0.14–0.95)∗
Male (ref)					
**Designation**					
Therapists	3.34 (1.12–10.09) ∗	2.71 (1.01–7.30)∗	0.84 (0.23–3.01)	0.36 (0.14–0.91)∗	1.08 (0.39–2.97)
Doctors and interns	1.51 (0.35–6.44)	7.27 (1.45–36.36)∗	15.88 (1.15–218.45)∗	3.34 (0.88–13.97)	0.17 (0.04–0.69)∗
Support Staff **(ref)**					
**Daily working hours**					
Up to 8 h	0.46 (0.12–1.73)	0.74 (0.18–3.13)	3.74 (0.52–26.89)	1.91 (0.47–7.83)	1.96 (0.48–8.00)
Greater than 8 h **(ref)**					
**Standing hours during work**					
Less than 4 h	0.37 (0.12–1.09)	0.64 (0.24–1.71)	0.34 (0.09–1.26)	0.95 (0.39–2.30)	1.66 (0.63–4.38)
Greater than 4 h (ref)					
**Sitting hours**					
Less than 4 h	0.44 (0.13–1.47)	0.82 (0.26–2.55)	0.79 (0.18–3.36)	1.62 (0.56–4.69)	0.76 (0.24–2.48)
Greater than 4 h (ref)					
**Break time at work**					
Up to 30 min per day	0.53 (0.21–1.37)	1.54 (0.61–3.87)	0.75 (0.22–2.54)	1.20 (0.51–2.84)	0.50 (0.19–1.33)
Greater than 30 min per day (ref)					
**Years of work experience**					
Up to 6 years	1.39 (0.48–4.02)	1.28 (0.44–3.69)	5.67 (1.38–23.18)∗	1.49 (0.55–3.99)	0.76 (0.27–2.16)
7 years and above (ref)					
**Obesity**					
Over weight	1.01 (0.35–2.92)	0.69 (0.24–2.01)	0.24 (0.05–1.08)	1.18 (0.46–3.04)	1.75 (0.65–4.70)
Obese	1.02 (0.36–2.76)	0.52 (0.19–1.37)	0.21 (0.05–0.91)∗	0.97 (0.41–2.30)	3.09 (1.19–8.03)∗
Underweight and normal (ref)					

Hosmer and Lemeshow test	0.668	0.530	0.952	0.994	0.979

The data are expressed as Odds ratio (95% CI). Burnout (ref-low; high); Pain (ref-no, yes); Any occupational symptoms (ref-no, yes); PCS (ref-low; high); MCS (ref-low, high); AOR = Adjusted odds ratio; CI= Confidence Interval, Binary logistic regression models were developed for each dependent variable to calculate adjusted odds ratios.

## 4 Discussion

This study, for the first time, explored the occupational health, general well-being, and quality of life among yoga and naturopathy health workers. The study included data on the characteristics, prevalence of ailments, and perceptions of naturopathic healthcare practitioners. According to the findings, the majority of the employees are female. This aligns with previous evidence indicating that females comprise 70% of the health and social care sector [11].

Almost a quarter of the employees were identified to be working for more than 8 h per day, which is almost identical to the prior statistics where the average working hour in a week is 46.9 h [12]. According to studies, working hours are related to burnout and can induce a variety of diseases such as depression, anxiety, sleep disorders, and coronary heart disease [12,13]. Therefore, reducing working hours is crucial for addressing employee well-being [14], and effective strategies on the subject are required even in a wellness setting. Furthermore, most health professionals reported sitting continuously for more than 4 h. Previous research has shown that prolonged sitting is linked to an increased risk of cardiometabolic and musculoskeletal problems [15,16], necessitating prompt action to incorporate some lifestyle counseling and some rejuvenation activities for the wellness staff as well.

The most common health issue among the study participants was pain. A cross-sectional study conducted in an Indian tertiary care hospital found a significant prevalence of work-related musculoskeletal disorders, especially low back pain, attributed to the nature of the profession, prolonged periods in the same position, and managing a large number of patients [17]. Naturopathy health care settings also demand for longer than usual consultation duration for doctors, as well as physically demanding duties for therapists such as delivering massage, hydrotherapy treatments, acupuncture, acupressure, and physiotherapy, and healthy meal, all of which may contribute to ergonomics imbalance and pain.

The study identified that 60% of the study participants had BMIs that were close to overweight or obesity. Additionally, there was a high level of burnout (29%), eye strain (41.3%) and sleep disruptions (26.8%) reported by the study participants. According to recent survey on naturopathic care, naturopaths primarily treat musculoskeletal, gastrointestinal, mental, and metabolic diseases using dietary changes, behavioral modifications, herbal therapy, and supplements [18].The current study, however, demonstrates that the population delivering naturopathic care are themselves dealing with such health difficulties. This may jeopardize the organization's/providers ability to provide high-quality care. So anticipatory plans addressing the health and well-being of naturopathic care providers are essential.

Another intriguing finding from the multivariate regression analysis was that females are more likely to experience pain, burnout, and occupational symptoms. This study adds to the evidence that women experience more pain than men, possibly due to hormonal differences, sex-related cortical differences in pain processing, differences in the endogenous opiate system, sociocultural beliefs about feminity and masculinity, and differences in pain coping mechanisms between men and women [19].

Another inference indicated that therapists were more likely to feel burnout, pain and poor PCS. This could be owing to the therapist's greater repetitive postures when providing naturopathic care. Doctors and interns were more likely to have pain, other occupational symptoms and poor MCS. This can be supported by literature which suggests that, doctors are more susceptible to mental health concerns such as anxiety, stress, and depression [20]. Furthermore, obesity was linked to lower MCS. Numerous studies have documented that individuals with obesity frequently experience higher levels of psychological distress, including anxiety and depression [21,22].

Although naturopathic doctors are trained to address health issues and are well-versed in prevntive measures and self-care practices, they still face mental health challenges. These may be attributed to long consultation hours, excessive paperwork, and extended screen time. Additionally, the prevalence of pain, burnout, obesity, and other work-related health issues among wellness providers suggests that many are not adhering to the necessary lifestyle changes and wellness practices. This could be due to extended working hours or a lack of employee-led wellness programs designed to promote occupational health in healthcare settings. However, this study did not conduct statistical analysis to substantiate these claims, which warrants further investigation in future research. Nevertheless, this underscores the importance of organizational policies and employee-driven wellness programs, such as stress management workshops, movement breaks, healthy snack options, sleep hygiene education, outdoor breaks, and ergonomic workspaces, to better support the health and well-being of the naturopathic workforce.

This study has some limitations. The cross-sectional study design itself could be considered a limitation as it may induce recall bias. Participants may not accurately remember or report past events or experiences, potentially affecting the reliability of the data collected. Additionally, the limited sample size restricts the generalizability of the findings, as the conclusions drawn may not be representative of the broader population of yoga and naturopathy health workers. Moreover, the data was collected from a single setting, which further limits the ability to generalize the results to other settings or populations. Future research with larger sample sizes and multiple study sites is recommended to validate and expand upon these findings.

## 5 Conclusion

In conclusion, this study provides valuable insights into the occupational health, general well-being, and quality of life among yoga and naturopathy health workers. Overall, by identifying specific occupational health challenges faced by yoga and naturopathy practitioners, this study underscores the necessity for targeted interventions and policies that prioritize the well-being of these professionals, ultimately fostering a healthier work environment and improving the quality of care they provide.

## Conflict of interest

None.

## Declaration of generative AI in scientific writing

None.

## Author contributions

PMK, KS, PBK, GRT: Conceptualization, Methodology; PMK, KS, PBK: Software; PMK, KS, PBK: Data curation, PMK, KS, PBK:Writing- Original draft preparation. PMK, KS: Visualization, Investigation. PMK, KS, GRT: Supervision.: PMK, KS, GRT: Software, Validation.: PMK, KS, PBK: Writing- Reviewing and Editing.

## Data statement

The data supporting the findings of this study are available from the corresponding authors upon reasonable request.

## Funding sources

This research did not receive any specific grant from funding agencies in the public, commercial, or not-for-profit sectors.
